# Targeting strategies in the treatment of fumarate hydratase deficient renal cell carcinoma

**DOI:** 10.3389/fonc.2022.906014

**Published:** 2022-07-15

**Authors:** Andrea Katharina Lindner, Gennadi Tulchiner, Andreas Seeber, Peter J. Siska, Martin Thurnher, Renate Pichler

**Affiliations:** ^1^ Department of Urology, Comprehensive Cancer Center Innsbruck, Medical University of Innsbruck, Innsbruck, Austria; ^2^ Department of Haematology and Oncology, Comprehensive Cancer Center Innsbruck, Medical University of Innsbruck, Innsbruck, Austria; ^3^ Department of Internal Medicine III, University Hospital Regensburg, Regensburg, Germany; ^4^ Immunotherapy Unit, Department of Urology, Medical University of Innsbruck, Innsbruck, Austria

**Keywords:** hereditary leiomyomatosis and renal cell cancer, renal cell carcinoma (RCC), metabolism, glucose, fumarate hydratase deficient renal cell carcinoma, bevacizumab, erlotinib, fumarate hydratase

## Abstract

Fumarate hydratase (FH) - deficient renal cell carcinoma (FHdRCC) is a rare aggressive subtype of RCC caused by a germline or sporadic loss-of-function mutation in the *FH* gene. Here, we summarize how FH deficiency results in the accumulation of fumarate, which in turn leads to activation of hypoxia-inducible factor (HIF) through inhibition of prolyl hydroxylases. HIF promotes tumorigenesis by orchestrating a metabolic switch to glycolysis even under normoxia, a phenomenon well-known as the Warburg effect. HIF activates the transcription of many genes, including vascular endothelial growth factor (VEGF). Crosstalk between HIF and epidermal growth factor receptor (EGFR) has also been described as a tumor-promoting mechanism. In this review we discuss therapeutic options for FHdRCC with a focus on anti-angiogenesis and EGFR-blockade. We also address potential targets that arise within the metabolic escape routes taken by FH-deficient cells for cell growth and survival.

## Renal cell carcinoma

Renal cell carcinoma (RCC) accounts for 2.6% of all cancers for almost 90% of malignant tumor formations of the kidney (1). The 2016 World Health Organization (WHO) Classification describes up to 14 known types of RCC, defining three main histological subtypes such as clear cell RCC (ccRCC), papillary RCC (pRCC) and chromophobe RCC (chRCC) (2). Additionally, a large spectrum of other renal tumors exists including a variety of uncommon, sporadic and familial cancers, of which about 15% are benign such as oncocytoma, metanephric tumors or angiomyolipoma (2). In ccRCC, chromosome 3p deletions are found in about 70% to 90% (3). Furthermore, inactivation of the *VHL* gene has been demonstrated in 100% of familial ccRCC and in 57% of sporadic ccRCC (4). Two subtypes of pRCC are known, type I and II, with type I tumors having better survival than type II (5). Chromosomal gain of chromosomes 7 and 17 are the most consistent and characteristic found genetic alteration (6). Originating from distal convoluted tubules, chRCC is characterized by multiple chromosomal losses of chromosomes Y, 1, 2, 6, 10, 13q, 17, and 21 (7). Metabolic reprogramming appears to be a key core aspect in the development and progression of RCC. The *VHL* gene is an important regulator that is often inactivated and consequently involves HIF activation, which in turn stimulates the reprogramming of several metabolic pathways. Reprogramming of the glucose, fat and amino acid metabolism dysregulate the tricarboxylic acid cycle leading to a subsequent pro-oncogenic cellular environment. Effective inhibitors or drugs reversing reprogrammed metabolic pathways is the basis of new and currently more emerging targeted therapies against RCC (8).

## RCC metabolism

Metabolism of glucose, amino acids and lipids is a key determinant of tumor growth (1 (9). RCC is a metabolically very active tumor. The key factors which are closely linked with tumor microenvironment (TME) metabolic alterations in RCC include HIF, fatty acid synthase (FASN) and pyruvate kinase 2 (PKM2), being potential targets in cancer therapy (10). Glycolysis and hypoxia play a key role in the failure of RCC therapy. ccRCC shows disturbed metabolism of glucose, amino acids and lipids (11, 12), driving tumor growth and affecting prognosis (13). Moreover, the significant feature of most ccRCCs is the loss of VHL, which causes HIF accumulation and drives the cellular hypoxic response. Consecutively, significant variations in metabolism have been found in ccRCCs (13, 14). In addition, molecular patterns involving metabolic pathways correlated with worse survival in ccRCC, including downregulation of AMP-activated kinase (AMPK) complex and the Krebs cycle genes, upregulation of genes involved in the pentose phosphate pathway and fatty acid synthesis (15). Functional deficiency of succinate dehydrogenase (SDH), resulting in succinate accumulation, is a common feature in up to 80% of ccRCCs (16). Survival analyses of the TCGA-KIRC dataset confirmed poor survival rates in case of lower expression of the SDH subunits SDHB, SDHC and SDHD (16). Notably, RCC tumors show a remarkable metabolic heterogeneity (17, 18), however, still little is known how specific cellular components of the TME contribute to therapy response or resistance mechanisms.

Modulation of metabolic ‘checkpoints’ driven by HIF and other factors may provide a new therapeutic strategy to reverse TME features responsible for RCC drug resistance. Metabolic changes including enhanced glycolysis and glutaminolysis as well as increased antioxidant activity have been associated with TKI resistance in RCC (19). The frequency of the metabolic reprogramming may render RCC a suitable disease for the investigation of potential novel therapeutic agents that target tumor metabolism.

## Fumarate hydratase deficient renal cell carcinoma (FHdRCC)

FHdRCC is a rare and aggressive subtype of type 2 papillary RCC, mainly affecting younger patients, metastazing early from small solitary lesions. It is caused by an inactivating mutation of the *FH* gene with a 15% lifetime risk for *FH* mutation carriers to develop RCC (20). Conversely, restoration of FH activity has been shown to stop development of FHdRCC and to restore the physiological metabolic phenotype in an animal model (21). FHdRCC can be associated with the hereditary leiomyomatosis and renal cell cancer (HLRCC) syndrome, an autosomal-dominant hereditable syndrome with predisposition to develop smooth muscle tumors of the skin and uterus. In the largest case series of HLRCC including 185 patients from 69 families, 12.4% developed RCC resulting in a lifetime risk of 21%. Although RCC occurs only in a minority of cases, it presents highly aggressive with poor survival in symptomatic patients with stage 3 to 4 disease. Thus, renal imaging screening should be offered to these patients resulting in earlier-stage diagnosis of RCC with consecutive survival benefit (20). The WHO lists the subtype ‘HLRCC-associated RCC’ in their RCC classification, which strictly speaking defines a group of genetically altered types of FHdRCC. The more general term FHdRCC should rather be used to include both hereditary and known sporadic forms of cancer development (22). FHdRCC shows histopathological features such as papillary architecture with tubule cystic growth patterns, abundant eosinophilic cytoplasm, perinucleolar halos and as shown recently, tumor cannibalism and lymphocytic emperipolesis (23). Yet, it still remains difficult to distinguish FHdRCC from papillary RCC by means of solely pathological criteria.

## Metabolic changes, epigenetic and signalling pathway alterations and therapeutic targets in FHdRCC

FH (EC 4.2.1.2) participates in the mitochondrial TCA cycle, which serves the production of cellular energy production in the form of ATP through oxidative phosphorylation (OXPHOS). FH catalyzes the conversion of fumarate to L-malate. Fumarate itself is the product of succinate oxidation by succinate dehydrogenase (16). The TCA cycle is fueled by acetyl-CoA, which condenses with oxaloacetate to form citrate in the first step of the pathway. To keep the TCA cycle going, acetyl-CoA must therefore continuously be generated either by oxidative decarboxylation of pyruvate, by oxidation of long-chain fatty acids, or by oxidative degradation of certain amino acids. When glucose levels are low or TCA cycle intermediates are diverted for biosynthetic purposes, cells use glutaminolysis to maintain the TCA cycle. In this pathway, glutaminase breaks down glutamine to form glutamate, which is further converted to α-ketoglutarate (α-KG). Of note, glutamine replenishment of the TCA cycle can result in fumarate accumulation even in FH-containing cells (24). An exciting study by Frezza *et al.* has addressed the mechanism that allows FH-deficient immortalized kidney cells to survive without a functional TCA cycle. By combining gas chromatography-mass spectrometry (GC-MS), liquid chromatography-mass spectrometry (LC-MS) and a computer model of metabolism, the authors found that FH-deficient cells used glutamine to survive and that accumulating fumarate was mainly glutamine-derived (24–26).

In a cataplerotic pathway, FH-deficient cells use the accumulated TCA cycle intermediate succinate to initiate porphyrin biosynthesis *via* succinyl-CoA. However, the resulting heme, i.e. iron protoporphyrin IX, is immediately degraded again by heme oxygenase (HMOX) and other enzymes. This apparently futile cycle of concomitant haem biosynthesis and degradation, enables FH-deficient cells to generate at least some mitochondrial NADH. The HMOX inhibitor zinc protoporphyrin or HMOX silencing impaired FH-deficient cell growth and colony formation. Likewise, inhibition of haem biosynthesis using hemin, an approved drug used for acute porphyria, reduced colony formation of FH-deficient cells, altogether indicating that cells lacking FH critically depend on this unusual pathway and that haem biosynthesis and degradation pathways may be attractive targets in the treatment of FHdRCC (24, 26). Yet another pathway that increases cellular levels of fumarate is the urea cycle, which converts toxic ammonia to urea for subsequent excretion. Ammonia mainly accumulates during amino acid catabolism. Fumarate is produced in the 4^th^ step of the cycle, when argininosuccinate is converted to arginine. Intriguingly, FH-deficient cells reverse this step and use fumarate and arginine to form argininosuccinate. Reversal of this metabolic step appears to be critical, since arginine depletion impaired cell proliferation and survival of FH-deficient cells (27). Therefore, arginine deprivation or targeting argininosuccinate synthase should be beneficial in this particular subtype of RCC (28).

It is not surprising that the lack of FH changes many cellular functions in FHdRCC. Fumarate and its immediate precursor succinate can act as oncometabolites by competitively inhibiting α-ketoglutarate (α-KG)-dependent dioxygenases, a family of enzymes that also includes prolyl hydroxylases. Suppression of protein prolyl hydroxylation slows down the degradation of HIF (21) and HIF stabilization in turn promotes tumorigenesis through transcriptional activation of pro-angiogenic genes including vascular endothelial growth factor (VEGF). HIF also activates glycolytic genes that contribute to the Warburg effect, a metabolic shift to aerobic glycolysis in normoxia. In addition to prolyl hydroxylase, fumarate and succinate inhibit other α-KG-dependent dioxygenases. Accumulating fumarate and succinate for instance suppress histone and DNA demethylases by depletion of α-KG causing genome-wide epigenetic modifications that further contribute to tumorigenesis (29). The mechanism of α-KG inhibition through accumulating fumarate is still debated, literature to date refers to this as a competitive inhibition process (30). For example, the histone de-methylation process is inhibited resulting in hypermethylation at histones H3K9 and H3K27 (31). Thus, transcription of tumor suppressor genes and differentiation genes is inhibited contributing to cell dedifferentiation, tumor progression and drug resistance (31). A TCGA pan-Kidney Cancer Analysis of 843 RCC confirmed distinctive features of each RCC subtype. FHdRCC was characterized by a CpG island methylator phenotype, DNA hypermethylation/CDKN2A alterations and increased immune signature expression for select immune gene signatures, including Th2 gene signature (similar to ccRCC) (32). In detail, poor outcome was linked in all RCC subtypes with hypermethylation of WNT pathway regulatory genes (*SFRP1* and *DKK1*), suggesting that hypermethylation of *SFRP1* and *DKK1* might be a promising prognostic biomarker in RCC (32). These observations may provide the therapeutic rationale of introducing immune checkpoint inhibitors, CDK4/6 inhibitors (33) and de-methylating agents in patients with FHdRCC.

In cancer cells with FH deficiency, fumarate can also react with cysteine residues of proteins in a non-enzymatic manner, generating S-(2-succinyl)cysteine. Such a protein modification, which is known as cysteine succination, eliminates the ability of Kelch-like ECH-associated protein 1 (KEAP1) to repress nuclear factor (erythroid-derived 2)-like 2 (NRF2) (34). Although activation of NRF2, a master regulator of antioxidant responses, in cancer can be beneficial, the fumarate-induced stabilization of NRF2 in HLRCC facilitates tumor growth and survival (34). SMARCC1 is a core member of the tumor suppressing SWI-SNF chromatin remodelling complex and also affected by succination. In ccRCC, SMARCC1 is commonly deleted because of its position on chromosome 3, which is known as a potentially tumor-promoting region in RCC. Using a fumarate-competitive chemoproteomic probe in concert with LC-MS, a recent study has identified a novel FH-regulated cysteine residue in SMARCC1 which is subject to succination (35). As a consequence, SWI-SNF complex formation is impaired and thus its tumor-suppressing activity is reduced.

In certain tumors, HIF stabilization and accumulation can also be induced by activation of epidermal growth factor receptor (EGFR) (36). EGFR signalling can increase the levels of HIF under normoxic conditions through the phosphoinositide 3-kinase (PI3K)/AKT pathway. EGFR can thus promote the Warburg effect. Consistent with EGFR-mediated HIF stabilization, EGFR inhibitors such as erlotinib can decrease the expression of the HIF target VEGF (37). Accordingly, resistance to EGFR inhibitors can be associated with increased levels of VEGF in the tumor microenvironment. Altogether, these observations have led to the concept of combining EGFR and VEGF(R) inhibitors (38). However, there are currently no reports about alterations of EGF signalling in FH-deficient tumors supporting the combined application of EGFR and VEGF(R) inhibitors.

The metabolic shift towards glycolysis in FHdRCC has been shown to lower the levels of AMPK (21). AMPK is a highly conserved metabolic sensor that governs cellular adaptation to energy deficiency and environmental stress. AMPK acts as a metabolic tumor suppressor by activating p53 and by regulating mammalian target of rapamycin (mTOR). The attenuation of AMPK in FHdRCC facilitates the activation of mTOR, which then promotes the biosynthetic pathways required for cell proliferation. Epidemiological studies indicate that the incidence of cancer is reduced in type 2 diabetes treated with the AMPK activator metformin (39). Therefore, pharmacological re-activation of AMPK using metformin would in principle also be a promising approach in the treatment of FHdRCC. However, metformin-induced AMPK activation occurs *via* inhibition of respiratory chain Complex I. By inhibiting mitochondrial OXPHOS, ATP is depleted, ultimately resulting in AMPK activation (40). Considering that FH deficiency itself causes a strong suppression of the mitochondrial respiration, it is currently unclear whether treatment with metformin would indeed be beneficial (26, 41).

## Clinical trials in HLRCC and FHdRCC

In metastatic FHdRCC, therapy regimens with immune checkpoint inhibitors (ICI), mTOR inhibitors, multi-target tyrosine kinase inhibitors (TKI) and various combinations have been tested in the past. PD-1/PD-L1 expression in tumor cells and tumor-infiltrating lymphocytes have been found only in a small proportion of FHdRCC cases. Since ICIs failed to induce satisfactory response rates, they should only be offered to patients with a PD-L1 positive tumor (42, 43). Dual inhibition of mTOR and VEGF was reported with ORR rates of up to 44%, whereas patients treated with mTOR inhibition alone showed no response (43, 44). TKIs were superior to mTOR inhibitors or ICIs alone, presenting with an ORR of up to 64% and a time to progression of 11.6 months (44). Cabozantinib, which is approved for metastatic RCC, might be the preferred TKI in HLRCC (45). In the AVATAR trial (NCT01130519), the combination of bevacizumab and erlotinib has shown promising preliminary results in HLRCC patients (46). In comparison to sporadic papillary RCC, the HLRCC cohort benefitted with an ORR of 72% and a median PFS of 21.1 months versus 35% and 8.8 months, respectively (46). Findings from a first-line setting of this combination revealed that FHdRCC patients treated with bevacizumab and erlotinib showed an ORR of 50% with a median PFS of 13.3 months and an impressive disease control rate of 90% (47). Pharmacological re-activation of AMPK using metformin may also be a promising approach in the treatment of FHdRCC. The combination of the VEGF/EGFR inhibitor vandetanib with metformin has already been tested in a phase II study (NCT02495103) but was not continued due to the lack of vandetanib availability. All registered studies in patients with FHdRCC are summarized in **Table 1**.

**Table 1:** Ongoing clinical trials in HLRCC and FHdRCC. .

Study number	Agents	Phase	Primary endpoint	Therapy setting	Status
**HLRCC**					
**NCT04981509**	bevacizumab + erlotinib + atezolizumab	II	CR	second line	recruiting
**NCT04603365**	pamiparib + temozolomide	II	ORR	second line	recruiting
**NCT02495103**	vandetanib + metformin	I/II	MTD/ORR	second line	terminated
**NCT01130519**	bevacizumab + erlotinib	II	ORR	first/second line	active, not recruiting
**FHdRCC**					
**NCT04068831**	talazoparib + avelumab	II	ORR	second line	recruiting
**NCT04387500**	sintilimab + axitinib	II	PFS, ORR	first line	recruiting
**NCT04146831**	sintilimab	II	PFS	second line	not yet recruiting

CR, complete response; ORR, objective response rate; PFS, progression-free survival; MTD, maximum tolerated dose. Status according to clinicaltrials.gov assessed on 08th February 2022.

## Outlook

**Figure 1:**
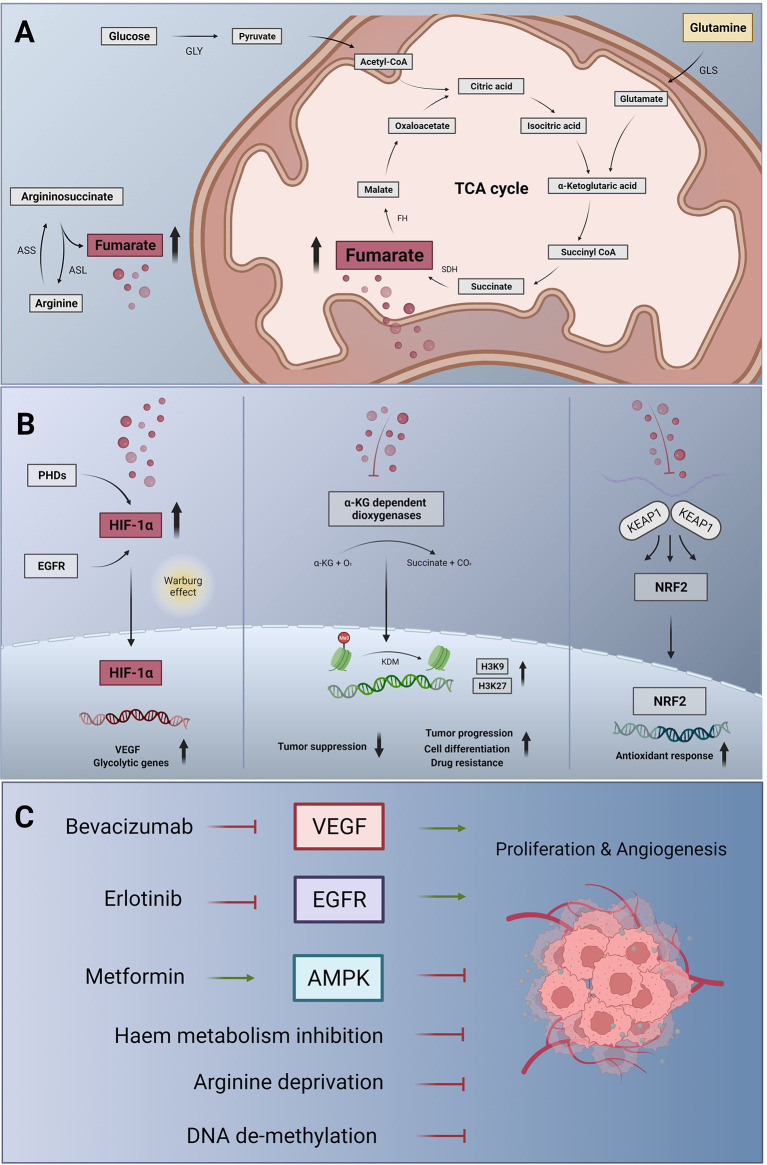
Metabolic changes, signalling pathway alterations and therapeutic targets in FHdRCC. **(A)** Metabolic changes in FH deficiency: in the glycolytic pathway, glucose is converted to pyruvate by glycolysis (GLY), which can enter the mitochondria and fuel the tricarboxylic acid cycle (TCA) cycle, also known as citrate as well as Krebs cycle. Succinate dehydrogenase (SDH) generates fumarate and fumarate hydratase **(FH)** catalyzes the stereospecific hydration of fumarate to form L-malate. Glutaminase (GLS) breaks down glutamine to form glutamate, which is further converted to α-ketoglutarate and feeds the TCA cycle. In the urea cycle, argininosuccinate is cleaved by argininosuccinase (ASL), producing additional fumarate. This step can be reversed by the argininosuccinase synthase (ASS) if argininosuccinate is required (27). In FH deficiency, fumarate accumulating in the mitochondria can leak out to the cytosol and become an ‘oncometabolite’. **(B)** Fumarate-induced activation of signalling pathways: cytosolic fumarate, like succinate, inhibits a family of prolyl hydroxylases (PHDs), which under normoxia destabilize hypoxia-inducible factor **(HIF)** through hydroxylation of prolyl residues. Fumarate (and succinate)-induced PHD inhibition causes HIF-1α accumulation. In the nucleus, HIF-1α activates the transcription of target genes including vascular endothelial growth factor (VEGF) and glycolytic genes, initiating the metabolic shift known as the Warburg effect (left panel). Fumarate and succinate accumulation can also act as α-ketoglutarate (α-KG) antagonist, inhibiting α-KG-dependent dioxygenases. Thus, histone de-methylation process catalyzed by histone demethylases (KDM) is inhibited. Consecutively, epigenetic alterations including hypermethylation at histone markers H3K9 and HRK27 inhibit tumor suppressor genes resulting in tumor progression, drug resistance and cell dedifferentiation (31, mid panel). In a non-enzymatic process, high concentrations of fumarate can also lead to the succination of cysteine residues of Kelch-like ECH-associated protein 1 (KEAP1), which thus loses its ability to prevent nuclear factor (erythroid-derived 2)-like 2 (NRF2)-mediated antioxidant responses (right panel). In FHdRCC, NRF2 activation is protumorigenic. **(C)** Targeting strategies for FHdRCC: fumarate-induced HIF activation promotes tumor angiogenesis and proliferation through VEGF signalling, suggesting the use of bevacizumab to neutralize VEGF. Activation of the epidermal growth factor receptor (EGFR), a frequent event in many tumors, can also activate HIF-1α, and HIF-1α induced VEGF can contribute to the resistance against EGFR inhibitor such as erlotinib. This has led to bevacizumab plus erlotinib combination therapy in certain cancer types including FHdRCC. The metabolic shift arising from FH deficiency results in decreased levels of adenosine monophosphate (AMP)-activated protein kinase (AMPK) and, as a consequence, of the tumor suppressor p53. Raising the activity of AMPK again may therefore also be desirable in FHdRCC, which can be achieved using metformin, an indirect AMPK activator (49). In addition, targeting the metabolic escape routes of FH-deficient cells through inhibitors of heme biosynthesis and degradation would be attractive in the treatment of FHdRCC (26). Given the strict arginine dependence of FH-deficient tumor cells arginine deprivation (27) and de-methylating agents (32) according to DNA CGI hypermethylation phenotype might be a therapy-supporting concept.

The future agenda may involve targeting of cancer cell metabolism at the level of glutaminolysis (48), to slow down TCA cycle activity and to avoid fumarate accumulation (**Figure 1**). The strict arginine dependence of FH-deficient cells also suggests arginine deprivation as an appropriate therapeutic approach (27). Moreover, OXPHOS, which generates ATP in a TCA cycle dependent manner, can be targeted using drugs such as metformin or possibly also atovaquone, and arsenic trioxide (50) to revert the tumorigenic metabolism through AMPK activation (50), provided that side effects are manageable. Gene therapy-based replacement of defective enzymes in target tissues, which is a current effort in the treatment of metabolic diseases (51), may be an ultimate goal help to improve the therapeutic opportunities of FH-deficient RCC. Moreover, targeting the epigenetic machinery to decrease the oncogenic impact of metabolic changes may be effective also in FHdRCC patients (31).

## Take home messages

In FHdRCC, the oncometabolite fumarate activates HIF-1α and promotes the tumorigenic Warburg effect. Anti-VEGF antibody bevacizumab plus EGFR inhibitor erlotinib showed response rates up to 72%. Targeting tumor metabolism aimed at AMPK re-activation may further improve FHdRCC therapy. Given the metabolic escape routes in FH-deficient cells, inhibition of haem biosynthesis and/or degradation as well as arginine deprivation offer novel therapeutic opportunities in FHdRCC. Additionally, DNA CpG islands (CGI) hypermethylation status might be a cornerstone of introducing de-methylating agents in the therapeutic landscape of FHdRCC.

## Conclusions

In conclusion, FHdRCC is a genetically or sporadically acquired aggressive variant of type 2 papillary kidney cancer that affects younger patients. Understanding the metabolic changes and signalling pathway alterations caused by FH deficiency may facilitate the development of more effective therapies for this aggressive and mostly fatal disease. Since the incidence of FHdRCC is low, it does not seem justified to screen all RCC patients for genetic abnormalities in the *FH* gene. However, it is important to raise awareness for clinical correlations and morphological signs to initiate further molecular and germline analysis. Long-lasting partial responses and manageable low-grade toxicities suggest that the combination therapy with bevacizumab and erlotinib should be considered as standard first-line therapy in locally advanced/metastatic FHdRCC.

## Author contributions

Conceptualization, AL, RP, and MT. Methodology, AL and GT. Data and literature curation, AL, GT, RP, MT, and AS. Writing—original draft preparation, AL, RP, and MT. Writing— review and editing, AS, GT, PS, and MT. Supervision, MT, PS, RP, and AS. Project administration, GT and AS. All authors have read and agreed to the published version of the manuscript.

## Conflict of interest 

The authors declare that the research was conducted in the absence of any commercial or financial relationships that could be construed as a potential conflict of interest.

## Publisher’s note

All claims expressed in this article are solely those of the authors and do not necessarily represent those of their affiliated organizations, or those of the publisher, the editors and the reviewers. Any product that may be evaluated in this article, or claim that may be made by its manufacturer, is not guaranteed or endorsed by the publisher.

## References

[1] Jemal A Murray T Ward E Samuels A Tiwari RC Ghafoor A. Cancer statistics, 2005. CA Cancer J Clin (2005) 55(1):10–30. doi: 10.3322/canjclin.55.1.10 15661684

[2] Moch H Cubilla AL Humphrey PA Reuter VE Ulbright TM. The 2016 WHO classification of tumours of the urinary system and Male genital organs-part a: Renal, penile, and testicular tumours. Eur Urol (2016) 70(1):93–105. doi: 10.1016/j.eururo.2016.02.029 26935559

[3] Cohen HT McGovern FJ. Renal-cell carcinoma. N Engl J Med (2005) 353(23):2477–90. doi: 10.1056/NEJMra043172 16339096

[4] Gnarra JR Tory K Weng Y Schmidt L Wei MH Li H. Mutations of the VHL tumour suppressor gene in renal carcinoma. Nat Genet (1994) 7(1):85–90. doi: 10.1038/ng0594-85 7915601

[5] Leroy X Zini L Leteurtre E Zerimech F Porchet N Aubert J-P. Morphologic subtyping of papillary renal cell carcinoma: correlation with prognosis and differential expression of MUC1 between the two subtypes. Mod Pathol (2002) 15(11):1126–30. doi: 10.1097/01.MP.0000036346.88874.25 12429790

[6] Brunelli M Eble JN Zhang S Martignoni G Cheng L. Gains of chromosomes 7, 17, 12, 16, and 20 and loss of y occur early in the evolution of papillary renal cell neoplasia: a fluorescent *in situ* hybridization study. Mod Pathol (2003) 16(10):1053–9. doi: 10.1097/01.MP.0000090924.90762.94 14559990

[7] Moch H Ohashi R. Chromophobe renal cell carcinoma: current and controversial issues. Pathology (2021) 53(1):101–8. doi: 10.1016/j.pathol.2020.09.015 33183792

[8] Chakraborty S Balan M Sabarwal A Choueiri TK Pal S. Metabolic reprogramming in renal cancer: Events of a metabolic disease. Biochim Biophys Acta Rev Cancer (2021) 1876(1):188559. doi: 10.1016/j.bbcan.2021.188559 33965513 PMC8349779

[9] Bian X Jiang H Meng Y Li Y-P Fang J Lu Z. Regulation of gene expression by glycolytic and gluconeogenic enzymes. Trends Cell Biol (2022) S0962-8924(22):00036–8. doi: 10.1016/j.tcb.2022.02.003 35300892

[10] Sun Q Chen X Ma J Peng H Wang F Zha X. Mammalian target of rapamycin up-regulation of pyruvate kinase isoenzyme type M2 is critical for aerobic glycolysis and tumor growth. Proc Natl Acad Sci USA (2011) 108(10):4129–34. doi: 10.1073/pnas.1014769108 PMC3054028 21325052

[11] Linehan WM Schmidt LS Crooks DR Wei D Srinivasan R Lang M. The metabolic basis of kidney cancer. Cancer Discov (2019) 9(8):1006–21. doi: 10.1158/2159-8290.CD-18-1354 31088840

[12] Du W Zhang L Brett-Morris A Aguila B Kerner J Hoppel CL. HIF drives lipid deposition and cancer in ccRCC *via* repression of fatty acid metabolism. Nat Commun (2017) 8(1):1769. doi: 10.1038/s41467-017-01965-8 29176561 PMC5701259

[13] Jing L Guigonis J-M Borchiellini D Durand M Pourcher T Ambrosetti D. LC-MS based metabolomic profiling for renal cell carcinoma histologic subtypes. Sci Rep (2019) 9(1):15635. doi: 10.1038/s41598-019-52059-y 31666664 PMC6821699

[14] Ou Y-C Li J-R Wang J-D Chang C-Y Wu C-C Chen W-Y. Fibronectin promotes cell growth and migration in human renal cell carcinoma cells. Int J Mol Sci (2019) 20(11):2792. doi: 10.3390/ijms20112792 PMC6600362 31181623

[15] Cancer Genome Atlas Research Network. Comprehensive molecular characterization of clear cell renal cell carcinoma. Nature (2013) 499(7456):43–9. doi: 10.1038/nature12222. PMC3771322 23792563

[16] Aggarwal RK Luchtel RA Machha V Tischer A Zou Y Pradhan K. Functional succinate dehydrogenase deficiency is a common adverse feature of clear cell renal cancer. Proc Natl Acad Sci USA (2021) 118(39):e2106947118. doi: 10.1073/pnas.2106947118 34551979 PMC8488664

[17] Zhang Y Udayakumar D Cai L Hu Z Kapur P Kho E-Y. Addressing metabolic heterogeneity in clear cell renal cell carcinoma with quantitative Dixon MRI. JCI Insight (2017) 2(15):e94278. doi: 10.1172/jci.insight.94278 PMC5543910 28768909

[18] Reinfeld BI Madden MZ Wolf MM Chytil A Bader JE Patterson AR. Cell-programmed nutrient partitioning in the tumour microenvironment. Nature (2021) 593(7858):282–8. doi: 10.1038/s41586-021-03442-1 PMC8122068 33828302

[19] Sato T Kawasaki Y Maekawa M Takasaki S Morozumi K Sato M. Metabolomic analysis to elucidate mechanisms of sunitinib resistance in renal cell carcinoma. Metabolites (2020) 11(1):1. doi: 10.3390/metabo11010001 33374949 PMC7821950

[20] Forde C Lim DHK Alwan Y Burghel G Butland L Cleaver R. Hereditary leiomyomatosis and renal cell cancer: Clinical, molecular, and screening features in a cohort of 185 affected individuals. Eur Urol Oncol (2020) 3(6):764–72. doi: 10.1016/j.euo.2019.11.002 31831373

[21] Tong W-H Sourbier C Kovtunovych G Jeong SY Vira M Ghosh M. The glycolytic shift in fumarate-hydratase-deficient kidney cancer lowers AMPK levels, increases anabolic propensities and lowers cellular iron levels. Cancer Cell (2011) 20(3):315–27. doi: 10.1016/j.ccr.2011.07.018 PMC3174047 21907923

[22] Lau HD Chan E Fan AC Kunder CA Williamson SR Zhou M. A clinicopathologic and molecular analysis of fumarate hydratase-deficient renal cell carcinoma in 32 patients. Am J Surg Pathol (2020) 44(1):98–110. doi: 10.1097/PAS.0000000000001372 31524643

[23] Billis A Assis-Mendonça GR Tavares TF Parreira K Costa LBE Barreto IS. Fumarate hydratase-deficient renal cell carcinoma: A tumor with diverse morphology including cannibalism, lymphocytic emperipolesis, and defective autophagy. Ann Diagn Pathol (2022) 56:151844. doi: 10.1016/j.anndiagpath.2021.151844 34753094

[24] Arts RJW Novakovic B Horst Rt Carvalho A Bekkering S Lachmandas E. Glutaminolysis and fumarate accumulation integrate immunometabolic and epigenetic programs in trained immunity. Cell Metab (2016) 24(6):807–19. doi: 10.1016/j.cmet.2016.10.008 PMC5742541 27866838

[25] Martínez-Reyes I Chandel NS. Mitochondrial TCA cycle metabolites control physiology and disease. Nat Commun (2020) 11(1):102. doi: 10.1038/s41467-019-13668-3 31900386 PMC6941980

[26] Frezza C Zheng L Folger O Rajagopalan KN MacKenzie ED Jerby L. Haem oxygenase is synthetically lethal with the tumour suppressor fumarate hydratase. Nature (2011) 477(7363):225–8. doi: 10.1038/nature10363 21849978

[27] Adam J Yang M Bauerschmidt C Kitagawa M O’Flaherty L Maheswaran P. A role for cytosolic fumarate hydratase in urea cycle metabolism and renal neoplasia. Cell Rep (2013) 3(5):1440–8. doi: 10.1016/j.celrep.2013.04.006 PMC3675675 23643539

[28] Delage B Fennell DA Nicholson L McNeish I Lemoine NR Crook T. Arginine deprivation and argininosuccinate synthetase expression in the treatment of cancer. Int J Cancer (2010) 126(12):2762–72. doi: 10.1002/ijc.25202 20104527

[29] Xiao M Yang H Xu W Ma S Lin H Zhu H. Inhibition of α-KG-dependent histone and DNA demethylases by fumarate and succinate that are accumulated in mutations of FH and SDH tumor suppressors. Genes Dev (2012) 26(12):1326–38. doi: 10.1101/gad.191056.112 PMC3387660 22677546

[30] Baksh SC Finley LWS. Metabolic coordination of cell fate by α-Ketoglutarate-Dependent dioxygenases. Trends Cell Biol (2021) 31(1):24–36. doi: 10.1016/j.tcb.2020.09.010 33092942 PMC7748998

[31] Tran TQ Lowman XH Kong M. Molecular pathways: Metabolic control of histone methylation and gene expression in cancer. Clin Cancer Res (2017) 23(15):4004–9. doi: 10.1158/1078-0432.CCR-16-2506 PMC5553983 28404599

[32] Ricketts CJ De Cubas AA Fan H Smith CC Lang M Reznik E. The cancer genome atlas comprehensive molecular characterization of renal cell carcinoma. Cell Rep (2018) 23(1):313–26. doi: 10.1016/j.celrep.2018.03.075 PMC6075733 29617669

[33] Sager RA Backe SJ Ahanin E Smith G Nsouli I Woodford MR. Therapeutic potential of CDK4/6 inhibitors in renal cell carcinoma. Nat Rev Urol (2022) 19(5):305–20. doi: 10.1038/s41585-022-00571-8 PMC9306014 35264774

[34] Adam J Hatipoglu E O’Flaherty L Ternette N Sahgal N Lockstone H. Renal cyst formation in Fh1-deficient mice is independent of the Hif/Phd pathway: roles for fumarate in KEAP1 succination and Nrf2 signaling. Cancer Cell (2011) 20(4):524–37. doi: 10.1016/j.ccr.2011.09.006 PMC3202623 22014577

[35] Kulkarni RA Bak DW Wei D Bergholtz SE Briney CA Shrimp JH. A chemoproteomic portrait of the oncometabolite fumarate. Nat Chem Biol (2019) 15(4):391–400. doi: 10.1038/s41589-018-0217-y 30718813 PMC6430658

[36] Peng X-H Karna P Cao Z Jiang B-H Zhou M Yang L. Cross-talk between epidermal growth factor receptor and hypoxia-inducible factor-1alpha signal pathways increases resistance to apoptosis by up-regulating survivin gene expression. J Biol Chem (2006) 281(36):25903–14. doi: 10.1074/jbc.M603414200 PMC3132567 16847054

[37] Pore N Jiang Z Gupta A Cerniglia G Kao GD Maity A. EGFR tyrosine kinase inhibitors decrease VEGF expression by both hypoxia-inducible factor (HIF)-1-independent and HIF-1-dependent mechanisms. Cancer Res (2006) 66(6):3197–204. doi: 10.1158/0008-5472.CAN-05-3090 16540671

[38] Naumov GN Nilsson MB Cascone T Briggs A Straume O Akslen LA. Combined vascular endothelial growth factor receptor and epidermal growth factor receptor (EGFR) blockade inhibits tumor growth in xenograft models of EGFR inhibitor resistance. Clin Cancer Res (2009) 15(10):3484–94. doi: 10.1158/1078-0432.CCR-08-2904 PMC2893040 19447865

[39] Luo Z Zang M Guo W. AMPK as a metabolic tumor suppressor: control of metabolism and cell growth. Future Oncol (2010) 6(3):457–70. doi: 10.2217/fon.09.174 PMC2854547 20222801

[40] Foretz M Guigas B Bertrand L Pollak M Viollet B. Metformin: from mechanisms of action to therapies. Cell Metab (2014) 20(6):953–66. doi: 10.1016/j.cmet.2014.09.018 25456737

[41] Tyrakis PA Yurkovich ME Sciacovelli M Papachristou EK Bridges HR Gaude E. Fumarate hydratase loss causes combined respiratory chain defects. Cell Rep (2017) 21(4):1036–47. doi: 10.1016/j.celrep.2017.09.092 PMC5668630 29069586

[42] Alaghehbandan R Stehlik J Trpkov K Magi-Galluzzi C Condom Mundo E Pane Foix M. Programmed death-1 (PD-1) receptor/PD-1 ligand (PD-L1) expression in fumarate hydratase-deficient renal cell carcinoma. Ann Diagn Pathol (2017) 29:17–22. doi: 10.1016/j.anndiagpath.2017.04.007 28807336

[43] Gleeson JP Nikolovski I Dinatale R Zucker M Knezevic A Patil S. Comprehensive molecular characterization and response to therapy in fumarate hydratase-deficient renal cell carcinoma. Clin Cancer Res (2021) 27(10):2910–9. doi: 10.1158/1078-0432.CCR-20-4367 PMC8127353 33658299

[44] Carril-Ajuria L Colomba E Cerbone L Romero-Ferreiro C Crouzet L Laguerre B. Response to systemic therapy in fumarate hydratase-deficient renal cell carcinoma. Eur J Cancer (2021) 151:106–14. doi: 10.1016/j.ejca.2021.04.009 33975058

[45] Martínez Chanzá N Xie W Asim Bilen M Dzimitrowicz H Burkart J Geynisman DM. Cabozantinib in advanced non-clear-cell renal cell carcinoma: a multicentre, retrospective, cohort study. Lancet Oncol (2019) 20(4):581–90. doi: 10.1016/S1470-2045(18)30907-0 PMC6849381 30827746

[46] Srinivasan R Gurram S Al Harthy M Singer EA Sidana A Shuch BM. Results from a phase II study of bevacizumab and erlotinib in subjects with advanced hereditary leiomyomatosis and renal cell cancer (HLRCC) or sporadic papillary renal cell cancer. JCO (2020) 38(15_suppl):5004. doi: 10.1200/JCO.2020.38.15_suppl.5004

[47] Choi Y Keam B Kim M Yoon S Kim D Choi JG. Bevacizumab plus erlotinib combination therapy for advanced hereditary leiomyomatosis and renal cell carcinoma-associated renal cell carcinoma: A multicenter retrospective analysis in Korean patients. Cancer Res Treat (2019) 51(4):1549–56. doi: 10.4143/crt.2019.086 PMC6790829 30913859

[48] Ganapathy-Kanniappan S Geschwind J-FH. Tumor glycolysis as a target for cancer therapy: progress and prospects. Mol Cancer (2013) 12:152. doi: 10.1186/1476-4598-12-152 24298908 PMC4223729

[49] Steinberg GR Carling D. AMP-activated protein kinase: the current landscape for drug development. Nat Rev Drug Discov (2019) 18(7):527–51. doi: 10.1038/s41573-019-0019-2 30867601

[50] Ashton TM McKenna WG Kunz-Schughart LA Higgins GS. Oxidative phosphorylation as an emerging target in cancer therapy. Clin Cancer Res (2018) 24(11):2482–90. doi: 10.1158/1078-0432.CCR-17-3070 29420223

[51] Kishnani PS Sun B Koeberl DD. Gene therapy for glycogen storage diseases. Hum Mol Genet (2019) 28(R1):R31–41. doi: 10.1093/hmg/ddz133 PMC6796997 31227835

